# How psychosocial outcomes impact on the self-reported health status in type 2 diabetes patients: Findings from the Diabetes Attitudes, Wishes and Needs (DAWN) study in eastern China

**DOI:** 10.1371/journal.pone.0190484

**Published:** 2018-01-25

**Authors:** Haijian Guo, Xuanxuan Wang, Tao Mao, Xiaoning Li, Ming Wu, Jiaying Chen

**Affiliations:** 1 School of Health Policy and Management, Nanjing Medical University, Nanjing, Jiangsu Province, China; 2 Jiangsu Provincial Center for Disease Control and Prevention, Nanjing, Jiangsu Province, China; 3 Creative Health Policy Research Group, Nanjing Medical University, Nanjing, Jiangsu Province, China; University of Rochester, UNITED STATES

## Abstract

**Introduction:**

The deleterious effects of psychosocial outcomes on diabetic patients’ health have not been fully investigated yet. This study was aimed to explore how psychosocial outcomes impacted on the health status of Chinese patients with type 2 diabetes.

**Methods:**

A mix of stratified sampling and typical sampling were used to select diabetic patients in Jiangsu Province to conduct individual interviews. Health status was measured by EuroQol Visual Analogue Scale (VAS). Psychosocial outcomes were measured by instruments used in the Diabetes Attitudes, Wishes and Needs survey, including psychological well-being, diabetes distress, patient empowerment, self-management, and patient reported healthcare provision. Clinical characteristics measured included diabetes comorbidities, complications and treatment. OLS regression analyses were used to estimate how health status varied with different characteristics.

**Results:**

Altogether 1614 patients with type 2 diabetes aged 18–65 years from 6 districts/counties in Jiangsu Province were included in the study. With general characteristics and clinical factors controlled for, psychological well-being among all psychosocial outcomes had the most significant association with health status, with a difference of 9.2 in VAS scores between likely depression and good well-being. VAS scores were also significantly lower in patients with high diabetes distress and significantly higher in those more frequently conducting physical activities. Other psychosocial outcomes were not significantly associated with health status.

**Conclusions:**

Likely depression and diabetes-related distress are negatively associated with health status while frequently conducting physical activities are positively associated with health status of type 2 diabetes patients aged 18–65 years from 6 districts/counties in Jiangsu Provinces. These findings underscore the necessity of undertaking routine assessment for depression and diabetes distress and prioritizing interventions on promoting regular physical activities in diabetic patients to improve health management and achieve better health outcomes.

## Introduction

Self-reported health status is a well-established quality of life measure for public health research and practice and is increasingly used as a key performance indicator in chronic illness [1, 2]. As one of the commonly used self-reported health status measures, the EuroQol Visual Analogue Scale (EQ-VAS) permits an overall health rating to be measured, which can capture the information on how patients with chronic illness perceive their health status [3].

Diabetes is one of the most common chronic diseases in nearly all countries, and will continue to rise in numbers and significance [4]. Diabetes also imposes heavy economic burden on the national healthcare system. In China, the health expenditure for diabetes among adults aged 20–79 was estimated to account for 6% of China’s total health expenditure in 2010 [5]. Given the growing epidemiological and economic burden of diabetes, it is necessary to identify the aspects that are substantially correlated with the self-rated health status of diabetic patients and have large room for improvement so as to set up targeted and effective interventions in the future.

There are overwhelming evidence on the deleterious effects of diabetes comorbidities and complications on the health status of diabetic patients [6, 7]. But in terms of non-clinical factors, the detrimental impact of psychosocial outcomes on diabetic patients has been underestimated [8].The Diabetes Attitudes, Wishes and Needs (DAWN) study is an internationally collaborative program, which was initiated in 2001 and launched again with a revised version in 2013. Investigating the psychosocial outcomes of patients with diabetes is one of its objectives [9]. The DAWN study confirmed that multiple psychosocial problems were common in diabetic patients but rarely assessed or treated [10, 11]. As a result, several international guidelines took the psychosocial outcomes of diabetes into account and formulated standards of psychological care for patients with diabetes, including the National Service Framework in UK [12], the International Diabetes Federation recommendations [13], and the American Diabetes Association guidelines [14]. In the Asian population, however, the psychosocial outcomes of patients with diabetes have not been explored with enough efforts yet. China was a participant country in the 2013 DAWN study, but to the best knowledge of the authors, no research has ever been conducted on the associations between psychological outcomes and the health status in Chinese diabetic patients.

This study was aimed to fill the knowledge gap of how psychosocial outcomes impacted on the self-rated health status of patients with type 2 diabetes in the Chinese population, with effects of clinical factors adjusted for.

## Methods

### Study design and sample

Data were drawn from a field survey of patients with type 2 diabetes in Jiangsu Province of China in 2016. Jiangsu Province is located at the eastern part of China. It is one of the first-batch pilot provinces to implement a comprehensive healthcare reform, which will be set as examples and provide experience for other provinces and municipalities.

A mix of stratified sampling and typical sampling were used to sample diabetic patients. In the first step, 6 districts (urban area) and counties (rural area) were selected, with 2 districts/counties located at the southern, middle and northern region of Jiangsu, respectively. All selected districts/counties were at the middle level of economic development within each region and had implemented tangible strategies of health management of diabetic patients, as suggested by experts from Jiangsu Provincial Center for Disease Control and Prevention. In the second step, 2 streets (urban area) and townships (rural area) in each district/county were sampled. All sampled streets were at the middle level of economic development and had similar population size. In the third step, 4 residential committees (urban area) and villages (rural area) in each street/township were sampled based on the same criteria as on the second step. Lastly, in each sampled residential/village, 100 diabetic patients were randomly selected from all patients registered in the diabetes health management system to conduct face-to-face structured interviews individually. The inclusion criteria were that patients should be at the age between 18 and 65 years old and were able to answer questions themselves. As clinical tests were also provided to participants before the survey and relevant clinical data were collected for other studies, the elderly may not have the capacity to complete all tests and questions. Therefore, patients above 65 years old were excluded.

Altogether 2474 diabetic patients participated in the survey, with 74 patients more than that required in the study plan. To avoid bias, the authors kept data of all participants. As this present study focused on patients with type 2 diabetes, 860 patients with type 1 diabetes, gestational diabetes and those who were not sure what type of diabetes they had were excluded from data analysis. All in all, 1614 patients with type 2 diabetes were included in the analysis.

### Data collection and quality of control

In each residential committee/village, a group of 8–10 doctors and nurses from the local community health centers (urban area) and township health centers (rural area) was formed to conduct the household survey. All interviewers had taken part in a training workshop held by two members of the research group before the survey to learn the objectives of the study, the survey questionnaire, and interpersonal interview techniques. The survey questionnaire contained questions on socioeconomic status, diabetes diagnosis and treatment, diabetes comorbidities and complications, psychosocial outcomes, diabetes-related health literacy and social support. Face-to-face individual interviews were carried out and each lasted for 30–40 minutes. At the end of the day, the research group members checked all questionnaires. If information was missing, the responsible interviewer called the corresponding patient at the same day or the next day to ask the question again.

### Ethical consideration

This study was approved by the Ethics Review Committee of Jiangsu Provincial Center for Disease Control and Prevention (No. JSJK2016-B003-03). Each potential interviewee was informed of the identity of the interviewer, the purpose of the interview, and the funding source. The interviewer also illustrated that the participation was anonymous and voluntary, and the interviewee could refuse to participate or terminate participation at any time during the interview without any influence on the health care services. The signed informed consent was obtained from each participant before the interview.

### Measurements

#### General characteristics

The age was divided into four groups: 18–40, 41–50, 51–60, and 61–65 years. The educational level was classified into five groups: below primary school, primary school, middle school, high school, and college and above. The marital status was categorized into single, married, and other status. The income level was divided into <1000 Chinese Yuan (1 Chinese Yuan ≈ US Dollar), 10000–30000 Chinese Yuan, 40000–50000 Chinese Yuan, 60000–100000 Chinese Yuan, and >1000000 Chinese Yuan.

#### Self-reported health status

EQ-VAS was used to measure self-reported health status in this present study, which had been tested and used in the 2008 National Health Services Survey of China [15]. EQ-VAS is a thermometer-like scale, on which the best health state one can imagine is marked 100 and the worst health state one can imagine is marked 0. Respondents were asked to point on the scale indicating how good or bad their health status was on the day when the survey was conducted [16].

#### Clinical characteristics

To identify diabetes comorbidities, diabetic patients were asked “Have you ever been diagnosed with the following diseases?” and a list of diabetes comorbidities could be chosen from, including hypertension, coronary heart disease, dyslipidemia, stroke, renal disease, malignant tumor, other comorbidity, and no comorbidity. Similarly, the question “Have you ever been diagnosed with the following diabetes complications?” was asked to diabetic patients and a list of diabetes complications could be chosen from, including diabetic foot, diabetic nephropathy, retinopathy, neuropathy, other complication, and no complication. In terms of diabetes treatment, the question “What is your current treatment for diabetes?” was asked and answering options included oral hypoglycemic agents, insulin, other treatment, and no treatment.

#### Psychosocial outcomes

Questionnaires used in the DAWN survey to measure psychological well-being, diabetes stress, patient empowerment, self-management and patient reported healthcare provision were applied in this present study. All questionnaires have been translated into the Chinese language, then back translated, and a harmonization process has been undertaken to ensure consistency with the original questionnaire [17]. The various measures were described below.

World Health Organization Well-being Index 5 (WHO-5) was used to measure psychological well-being. There are five statements following the question “Please indicate for each of the five statements, which is closet to how you have been feeling over the last 2 weeks”, which are “I have felt cheerful and in good spirits”, “I have felt calm and relaxed”, “I have felt active and vigorous”, “I woke up feeling fresh and rested”, and “My daily life has been filled with things that interest me.”. There are six response options for each statement: “all of the time”, “most of the time”, “more than half of the time”, “less than half of the time”, “some of the time”, and “at no time”. Respondents were asked to select one response for each statement. A comprehensive score for WHO-5 can be calculated ranging from 0–100. Scores of ≤ 28 indicate likely depression, between 29 and 50 refer to reduced well-being, and > 50 denote good well-being [16].

Problem Areas in Diabetes Scale 5 (PAID-5) was used to measure diabetes-related stress. Respondents were asked the question “Which of the following diabetes issues are currently a problem for you?” and then invited to select one response for each diabetes issue that gives the best answer. The five diabetes issues are “Feeling scared when you think about living with diabetes”, “Feeling depressed when you think about living with diabetes”, “Worrying about the future and the possibility of serious complications”, “Feeling that diabetes is taking up too much of your mental and physical energy every day”, and “Coping with complications of diabetes”. The response options are “not a problem”, “minor problem”, “moderate problem”, “somewhat serious problem”, and “serious problem”. A comprehensive score for PAID-5 can be calculated ranging from 0–100. Scores of ≥ 40 indicate high diabetes-related distress [16].

Diabetes Empowerment Scale-DAWN Short Form (DES-DSF) was used to measure patients’ confidence in taking an active role in their own management of conditions. The five statements following the question “How often do you do the following?” are “Let people know how they can best support you in managing your diabetes”, “Try out different ways to better manage your diabetes”, “Ask for support to help manage your diabetes when you need it”, “Seek out the information you need to manage your diabetes”, and “Take part in activities in your community to improve care for people with diabetes”. There are five response options for each statement: “never”, “rarely”, “sometimes”, “often”, and “always”. Respondents were asked to select one response for each statement. A comprehensive score for DES-DSF can be calculated ranging from 0–100. Higher scores indicate higher levels of patient empowerment [16, 18].

Summary of Diabetes Self-Care Activities (SDSCA) was used to measure patient’s self-care activities. Respondents were asked to think how many of the last seven days before the survey on which they performed such diabetes self-care activities. There are six self-management activities, which are “Have you followed a healthy eating plan?” “Did you participate in at least 30 min of physical activity?” “Did you test your blood sugar?” “Did you test your blood sugar the number of times recommended by your healthcare provider?” “Did you check your feet” “Did you take all your diabetes medications exactly as agreed with your healthcare professional?” Each item is treated individually. Another question “Did you smoke during the last seven days?” was included in the measure and patients needed to choose the answer from “yes” and “no” [16].

Patient Assessment of Chronic Illness Care-DAWN Short Form (PACIC-DSF) was used to measure how patient perceived the support from the healthcare team. Patients were asked to choose from five response options–“none of the time”, “a little of the time”, “some of the time”, “most of the time”, and “always”–for each statement to assess how frequently they could receive the specific healthcare. Following the ingress “Over the past 12 months, when I received care for my diabetes”, there are 12 statement, which are “I was asked how my diabetes affects my life”, “I was asked to talk about any problems with my medicines or their effects”, “I was asked for my ideas when a plan was made for my diabetes care”, “My healthcare team encouraged me to ask questions”, “My healthcare team listened to how I would like to do things”, “I was helped to set specific goals to improve the management of my diabetes”, “I was helped to make plans to achieve my diabetes care goals”, “My healthcare team conveyed confidence in my ability to make changes”, “I was helped to make plans for how to get support from my friends, family or community”, “I was encouraged to go to a specific group or class to help me cope with my diabetes”, “I was contacted after a visit to see how things were going”, and “I was satisfied that my care was well organized”. A comprehensive score for PACIC-DSF can be calculated ranging from 1–5. Higher scores refer to higher patient satisfaction [16, 18].

#### Statistical analyses

Descriptive analyses were performed on sex, age group, socioeconomic status, clinical characteristics, self-reported health status and psychosocial outcomes. OLS regression analyses were used to estimate how EQ VAS scores varied with different characteristics. Three regression models were built. In the first model, only variables of sex, age group and socioeconomic status were included. In the second model, variables of clinical characteristics were added. In the last model, variables of psychosocial outcomes were added. Dummy variables were created for age group, education level, marital status, income level, diabetes comorbidities, diabetes complications, diabetes treatment, and psychological well-being. A *p* value<0.05 was considered for statistical significance throughout the analyses.

## Results

As shown in Table 1, male and female diabetic patients were of the same proportion. The elderly aged between 51 and 65 years old occupied nearly 70% of participants. More than half of the participants had an education level of middle school or above. The majority of diabetic patients in this study got married (93.6%). Nearly 40% diabetic patients had annual household income of 10000–30000 Chinese Yuan, followed by 40000–50000 Chinese Yuan (21.5%) and 60000–100000 Chinese Yuan (16.3%), and only around 8% participants were at the highest income group.

**Table 1:** General characteristics of type 2 diabetes patients in eastern China (N = 1614).

Variables	n	%
**Overall**	1614	100.0
**Sex**		
Men	800	49.6
Women	814	50.4
**Age**		
Mean ± SD	53.7± 8.9	
18–40	141	8.7
41–50	368	22.8
51–60	677	41.9
61–65	428	26.5
**Education level**		
Below primary school	294	18.2
Primary school	392	24.3
Middle school	636	39.4
High school	264	16.4
College and above	28	1.7
**Marital status**		
Single	39	2.4
Married	1511	93.6
Other status	64	4.0
**Income level**		
Income < 10000 Chinese Yuan	249	15.4
10000≤ Income <40000 Chinese Yuan	617	38.2
40000≤ Income < 60000 Chinese Yuan	347	21.5
60000≤ Income <100000 Chinese Yuan	263	16.3
Income≥ 100000 Chinese Yuan	138	8.6

As demonstrated in Table 2, more than 60% diabetic patients in the survey reported having diabetes comorbidities. Hypertension was the most prevalent comorbidity (45.2%), followed in sequence by dyslipidemia (22.2%), other comorbidity (10.5%), coronary heart disease (4.6%), stroke (3.4%), renal disease (2.2%), and malignant tumor (1.2%). Less than 30% of all respondents in the survey reported having diabetes complications. Retinopathy, accounting for 21.6%, was the most prevalent complication, followed in sequence by diabetic foot (7.1%), neuropathy (6.2%), diabetic nephropathy (4.5%), and other complication (0.9%). In the survey, oral hypoglycemic agents were reported as the most common treatment for type 2 diabetes (74.4%). Those who took insulin accounted for 16.4% of participants. About 13% diabetic patients reported that they did not take any treatment.

**Table 2:** Clinical characteristics of type 2 diabetes patients in eastern China (N = 1614).

Variables	n	%
**Diabetes comorbidities**		
Hypertension	730	45.2
Coronary heart disease	75	4.6
Dyslipidemia	358	22.2
Stroke	55	3.4
Renal disease	35	2.2
Malignant tumor	20	1.2
Other comorbidity	170	10.5
No comorbidity	616	38.2
**Diabetes complications**		
Diabetic foot	115	7.1
Diabetic nephropathy	72	4.5
Retinopathy	348	21.6
Neuropathy	100	6.2
Other complication	15	0.9
No complication	1178	73.0
**Diabetes treatment**		
Oral hypoglycemic agents	1201	74.4
Insulin	264	16.4
Other treatment	17	1.1
No treatment	217	13.4

In this study, the self-reported health status of diabetic patients was prone to be good, with an average EQ VAS score of 75.8 (Table 3). The majority of diabetic patients demonstrated good psychological well-being, with 87.1% having a WHO-5 score above 50. Reduced psychological well-being and likely depression were found in 7.5% and 5.4% of participants, respectively. In terms of diabetes distress, 44.9% diabetic patients exhibited high diabetes-related distress. Patients’ empowerment in their own management of diabetes was at the middle level, with an average score of 53.2 on a 0–100 scale. The most common self-care activity among diabetic patients during the past 7 days before the survey was keeping a healthy diet. On average, in 5 out of 7 days diabetic patients could take all their diabetes medications exactly as agreed with their healthcare professionals. Less common self-care activity was participating in physical activity, followed by testing blood sugar the number of times recommended by the healthcare provider, checking the feet, and testing glucose generally. During the past 7 days before the survey, 77% participants did not smoke. As to healthcare provision, in general, diabetes-related care provided by healthcare professionals could be perceived by patients for some of the time, with an average PACIC-DSF score of 3.3.

**Table 3:** Self-reported health status, psychological well-being, diabetes distress, empowerment and healthcare provision of type 2 diabetes patients in eastern China (N = 1614).

Variables	n	%
**Self-reported health status**		
EQ VAS (Mean ± SD)	75.8 ± 16.2	
**Psychological well-being**		
WHO-5 (Mean ± SD)	76.2± 22.5	
WHO-5 ≤ 28	87	5.4
29 ≤ WHO-5 ≤ 50	121	7.5
WHO-5 > 50	1406	87.1
**Diabetes distress**		
PAID-5 (Mean ± SD)	36.9 ± 26.9	
PAID-5 < 40	889	55.1
PAID-5 ≥ 40	725	44.9
**Patient empowerment**		
DES-DSF (Mean ± SD)	53.2 ± 18.5	
**Self-management** (Mean ± SD)		
Healthy diet	5.5 ± 2.2	
Physical activity	3.8 ± 2.9	
SMBG	2.2 ± 2.5	
SMBG as recommended	2.8 ± 2.7	
Feet exam	2.3 ± 2.8	
Drug assumption as recommended	5.0 ± 2.8	
Smoking		
Yes	371	23.0
No	1243	77.0
**Healthcare provision**		
PACIC-DSF (Mean ± SD)	3.3 ± 1.1	

EQ-VAS: EuroQol Visual Analogue Scale; WHO-5, World Health Organization Well-Being Index 5; PAID-5, Problem Areas in Diabetes Scale 5; DES-DSF, Diabetes Empowerment Scale-DAWN Short Form; SMBG, Self-Monitoring of Blood Glucose; PACIC-DSF, Patient Assessment of Chronic Illness Care-DAWN Short Form.

In the first regression model, sex, age group and socioeconomic status were included (Table 4). The difference in VAS scores between male and female diabetic patients was not significant. The VAS scores declined significantly with age, the difference between the age group of 18–40 years and the group of 51–60 years being 4.6 scores. The effect of being at the oldest age group was not significant. The VAS scores were significantly higher for higher levels of education, but this education gradient was clear for participants with the middle school education level and above, with a difference of 6.9 between the highest and lowest education level. No significant differences were found between different groups of marital status. In terms of income, the VAS scores were significantly higher in higher income groups, the difference between the highest and lowest income group being 11.0.

**Table 4:** Impacts of general characteristics, clinical characteristics, psychological well-being, diabetes distress, empowerment, self-management and healthcare provision on self-reported health status among type 2 diabetes patients in eastern China (N = 1614).

Variables	Model 1		Model 2		Model 3	
	*β*	*p*	*β*	*p*	*β*	*p*
**Constant**	73.852	0.000	78.444	0.000	80.025	0.000
**Sex** ^a^						
Women	-0.541	0.533	-0.923	0.261	-0.670	0.462
**Age group** ^b^						
41–50	-3.311	0.040	-1.266	0.414	-1.004	0.509
51–60	-4.601	0.002	-1.786	0.228	-2.048	0.158
61–65	-3.072	0.061	-0.129	0.936	-0.690	0.661
**Education level** ^c^						
Primary school	-0.151	0.905	-1.083	0.367	-2.155	0.068
Middle school	2.640	0.038	0.780	0.522	-0.287	0.811
High school	4.906	0.001	2.630	0.072	1.542	0.286
College and above	6.946	0.034	7.332	0.019	5.054	0.100
**Marital status** ^d^						
Married	-1.247	0.641	-1.056	0.678	-1.673	0.501
Other status	-2.345	0.475	-2.342	0.452	-2.434	0.424
**Income level** ^e^						
10000≤ Income <40000 Chinese Yuan	4.461	0.000	3.470	0.003	2.351	0.041
40000≤ Income < 60000 Chinese Yuan	5.798	0.000	3.758	0.005	2.068	0.115
60000≤ Income <100000 Chinese Yuan	7.386	0.000	5.064	0.000	3.440	0.013
Income ≥ 100000 Chinese Yuan	11.027	0.000	8.276	0.000	5.944	0.000
**Diabetes comorbidities** ^f^						
Hypertension	-	-	-1.243	0.321	-1.312	0.285
Coronary heart disease	-	-	-5.437	0.003	-4.336	0.016
Dyslipidemia	-	-	-4.305	0.000	-4.116	0.000
Stroke	-	-	-7.401	0.000	-6.098	0.003
Renal disease	-	-	-9.233	0.001	-8.051	0.003
Malignant tumor	-	-	-6.696	0.052	-6.347	0.060
Other comorbidity	-	-	-4.762	0.002	-4.190	0.005
No comorbidity ^g^	-	-	-0.308	0.835	-0.122	0.933
**Diabetes complications** ^h^						
Diabetic foot	-	-	1.169	0.507	0.215	0.901
Diabetic nephropathy	-	-	-0.310	0.883	-0.490	0.812
Retinopathy	-	-	-3.043	0.119	-2.449	0.201
Neuropathy	-	-	-1.845	0.298	-0.035	0.984
Other complication	-	-	-4.566	0.275	-4.868	0.234
No complication ^i^	-	-	4.353	0.035	4.135	0.041
**Diabetes treatment** ^j^						
Oral hypoglycemic agents	-	-	-3.645	0.059	-2.212	0.242
Insulin	-	-	-3.924	0.022	-3.084	0.066
Other treatment	-	-	-6.326	0.100	-4.078	0.282
No treatment ^k^	-	-	-2.523	0.258	-2.143	0.349
**Psychological well-being** ^l^						
WHO-5 ≤ 28	-	-	-	-	-9.190	0.000
29 ≤ WHO-5 ≤ 50	-	-	-	-	-7.679	0.000
**Diabetes stress** ^m^						
PAID-5 ≥ 40	-	-	-	-	-2.081	0.007
**Patient empowerment**						
DES-DSF	-	-	-	-	0.031	0.176
**Self-management**						
Healthy diet	-	-	-	-	-0.303	0.108
Physical activity	-	-	-	-	0.483	0.001
SMBG	-	-	-	-	-0.320	0.156
SMBG as recommended	-	-	-	-	0.357	0.083
Feet exam	-	-	-	-	-0.047	0.745
Drug assumption as recommended	-	-	-	-	-0.252	0.144
Smoking ^n^	-	-	-	-	0.161	0.872
**Healthcare provision**						
PACIC-DSF	-	-	-	-	0.250	0.496
**R Square**	0.069		0.181		0.227	

^a^ Reference category: men.

^b^ Reference category: age group 18–40 years.

^c^ Reference category: below primary school.

^d^ Reference category: single.

^e^ Reference category: income level < 10000 RMB.

^f^ Coded: having such comorbidity = 1; not having such comorbidity = 0.

^g^ Coded: having no comorbidity = 1; having any one comorbidity = 0.

^h^ Coded: having such complication = 1; not having such complication = 0.

^i^ Coded: having no complication = 1; having any one complication = 0.

^j^ Coded: using such treatment = 1; not using such treatment = 0.

^k^ Coded: using no treatment = 1; using any treatment = 0.

^l^ Reference category: WHO-5 > 50.

^m^ Reference category: PAID-5 < 40.

^n^ Reference category: smoking.

WHO-5, World Health Organization Well-Being Index 5; PAID-5, Problem Areas in Diabetes Scale 5; DES-DSF, Diabetes Empowerment Scale-DAWN Short Form; SMBG, Self-Monitoring of Blood Glucose; PACIC-DSF, Patient Assessment of Chronic Illness Care-DAWN Short Form.

In the second model, clinical characteristics of diabetic patients were added. Participants who had been diagnosed with renal disease had a 9.2 lower VAS score than those who had not been diagnosed with such disease. The VAS scores were also significantly lower in participants with stroke, coronary heart disease, other comorbidity, and dyslipidemia compared to those without such diseases. The difference in VAS scores between participants with a certain type of diabetes complication and those without was not significant. However, the VAS scores were 4.4 higher for participants reporting having no diabetes complication compared to those having at least one type of diabetes complication. The VAS scores were 3.9 lower for diabetic patients taking insulin compared to those not using insulin.

In the last model, psychosocial outcomes were entered. The gradient of psychological well-being was clear, with a difference of 9.2 between likely depression (WHO-5 ≤ 28) and good well-being (WHO-5 > 50). At the same time, the VAS scores were significantly lower in diabetic patients with high diabetes-related distress, with a difference of 2.1 between patients with high diabetes distress (PAID-5 ≥ 40) and those with a PAID-5 score below 40. Among all self-care activities, only participating in physical activity had a significantly positive association with the self-reported health status. Neither patient empowerment nor healthcare provision was significantly associated with the self-reported health status in this study.

## Discussion

To the best knowledge of the authors, this study is the first to examine how psychosocial outcomes impact on the self-reported health status among Chinese diabetic patients with clinical characteristics controlled for. Our findings demonstrate that among Chinese patients with type 2 diabetes, likely depression, reduced psychological well-being, high diabetes-related distress and having diabetes comorbidities, particularly renal disease, malignant tumor and stroke, are associated with poorer self-reported health status, while more frequently conducting physical activities and having no diabetes complications are associated with better self-reported health status.

In this study, the negative effect of poor psychological well-being was significant. About 5% and 8% diabetic patients had likely depression (WHO-5 ≤ 28) and reduced psychological well-being (29 ≤ WHO-5 ≤ 50) respectively, whose EQ-VAS decreased by more than 9 and 7 scores respectively with clinical characteristics and other psychosocial outcomes controlled for, compared to those had a WHO-5 score over 50. The prevalence of diabetes-related distress was quite high in this study, with nearly 45% diabetic patients having a PAID-5 score above 40. In consistency with previous studies, our findings confirm that depression and diabetes-related distress are prominent psychological problems in diabetic patients [19, 20]. As routine assessment for depression and diabetes distress has not been required in the health management of patients with type 2 diabetes in China yet, it is recommended that such services should be provided to diabetic patients in order to improve the health management and patients’ health status. Through routine use of reliable and less time-consuming tools such as WHO-5 and PAID-5, detecting and monitoring depression and diabetes distress in diabetic patients could be realized so as to design targeted treatment regimen and achieve better health outcomes [21]. In Germany, WHO-5 has already been advised in the national guideline of diabetes care as the specific screening tool for depression due to its simplicity, easy applicability and rapid evaluation [22].

Although both WHO-5 and PAID-5 are validated instruments used to measure psychological disorders in diabetic patients, depression and diabetes distress contain distinctly different psychological constructs [23]. WHO-5 is used to assess general psychological well-being and determine depression [24], and PAID-5 is designed to track the emotional burden of diabetes by evaluating how diabetes management and feelings about diabetes are to the diabetic patients [25]. As the results shown in this present study, the prevalence of depression was lower than that of diabetes distress among Chinese patients with type 2 diabetes, while depression measured by WHO-5 had a much stronger negative correlation with the self-reported health status than diabetes distress measured by PAID-5 did. These results suggested that both instruments should be used in the health management of Chinese diabetic patients in order to determine depression and identify the most distressed aspects of diabetes.

In terms of self-care activities, the results show that in Chinese patients with type 2 diabetes, conducting physical activities more frequently is more likely to perceive better health status. Similar results have been observed in previous studies [26]. Regular physical activity has been proven to be conducive to promoting glycemic control, enabling better blood glucose control, and improving glycaemia by lowering insulin resistance [27, 28]. Findings in this present study contribute to the current body of knowledge by demonstrating that the benefits of performing physical activities on health outweigh those of the other six self-care behaviors. Hence, it is recommend that interventions on promoting regular physical activities among patients with type 2 diabetes in China should be prioritized.

Several limitations of this present study should be addressed. Firstly, no data on diabetic patients aged over 65 years old were collected in the survey. Although the elderly were excluded because they may not be able to complete all the clinical tests and the 40-minute survey, it would be of great value to learn their psychosocial outcomes and self-reported health status. Such information should be collected and analyzed in future research. Secondly, due to the cross-sectional study design, no causal relations can be determined between psychosocial outcomes and the self-reported health status. Thirdly, Jiangsu is a relatively prosperous province in China and the six selected districts/counties in this study have implemented more tangible health management of diabetic patients than the other areas in Jiangsu Province; therefore, the results of this present study cannot be generalized. But as Jiangsu is one of the four pilot provinces to implement a comprehensive healthcare reform, this study might provide references for similar studies conducted in other parts of China. Future research could use follow-up data to monitor the change in psychosocial outcomes and identify the determinant factors of self-reported health status in Chinese diabetic patients.
